# A Discourse on the Phenomena of Sensation, as Connected with the Mental, Physical, and Instinctive Faculties of Man

**Published:** 1841-04

**Authors:** 


ART. I.—*A Discourse on the Phenomena of Sensation, as connected with the Mental, Physical, and Instinctive Faculties of Man.* By JAMES JOHNSTONE, M.D., Physician to the General Hospital, Birmingham, &c. —*London*, 1841. 8vo, pp. 264.

THIS treatise, as we are informed by its author, contains the substance of a course of Lectures delivered by him in the spring of 1838; but what was his motive for presenting it to the public, he has not stated; and we find ourselves rather at a loss to account for its appearance. Medical books may be distributed under two classes; those which are the result of an honest conviction on the part of their writers, that they are in the possession of knowledge, (whether this be acquired by original research, or by collecting and comparing the statements of others) which may be advantageously diffused amongst their brethren; and those which are the fruit of a desire to make themselves known as authors, on account of certain advantages which they expect to derive, either in the shape of literary or scientific fame, or of profitable reputation with the public. Dr. Johnstone's character and position in the medical world forbid us to impute to him any selfish motive; and yet we are surprised that he is so far ignorant of medical literature as to suppose that his treatise contains anything either new in itself, or new in its manner of production. It is in fact a mere compilation, which might very well serve as part of a course of lectures, but of which a very small amount of information on the part of its readers would enable them to perceive the sources. As a purely elementary treatise, it may be put into the hands of the student who is commencing the pursuit of physiology; but we must qualify this recommendation by observing that we do not consider that any single system can be advantageously treated of in such an isolated manner, unless an amount of detail respecting its relations to other systems be given, which shall render it complete in itself. This is very far from being the case in Dr. Johnstone's discourse, which is, moreover, not only imperfect in this respect, but much behind the present state of knowledge in almost every department of the subject. Such hypotheses as the following are worse than useless in an elementary treatise: "I do not mean to assert that nervous influence and electricity are absolutely identical; but it is surely far from impossible that the former may be a subtile modification of the latter." Further on, we find that "there are two kinds of sensation, the one of which is unaccompanied with consciousness, and which, for the sake of distinction, we shall call *negative* sensation; while the other, which is attended with consciousness, we shall name *positive* sensation." We must here again protest against the confusion of ideas that the employment of the term *sensation* to designate any change in which consciousness is not involved is certain to produce. In the chapter on Involuntary Motions, we find the *dependence* of the heart's action and of the peristaltic movements of the alimentary canal upon the ganglionic system, strongly advocated—a doctrine which we regard as overthrown by the latest and best experimental enquiries on the properties of muscular fibre. And in the next chapter, we find the actions excited through the spinal system of nerves designated as *semi-voluntary*, although a very little consideration will show that the will does not in the least degree participate in their production, although they are in various degrees controlled and directed by it. If Dr. Johnstone will hold his breath for a few moments he will find that no effort of his will can prevent respiratory actions; nor will he be able by a voluntary effort to avoid performing the movements of deglutition when the requisite stimulus is applied. So many similar instances of loose and inaccurate generalization occur in the treatise, that we are obliged to regret (which we do most sincerely) our inability to pronounce a more favorable opinion on it.

